# Somatosensory and transcranial motor evoked potential monitoring in a porcine model for experimental procedures

**DOI:** 10.1371/journal.pone.0205410

**Published:** 2018-10-08

**Authors:** Sven Maier, Ulrich Goebel, Sonja Krause, Christoph Benk, Martin A. Schick, Hartmut Buerkle, Friedhelm Beyersdorf, Fabian A. Kari, Jakob Wollborn

**Affiliations:** 1 Department of Cardiovascular Surgery, University Heart Center Freiburg, Freiburg, Germany; 2 Faculty of Medicine, University of Freiburg, Freiburg, Germany; 3 Department of Anesthesiology and Critical Care, Medical Center–University of Freiburg, Freiburg, Germany; Albert Einstein College of Medicine, UNITED STATES

## Abstract

Evoked potential monitoring has evolved as an essential tool not only for elaborate neurological diagnostics, but also for general clinical practice. Moreover, it is increasingly used to guide surgical procedures and prognosticate neurological outcome in the critical care unit, e.g. after cardiac arrest. Experimental animal models aim to simulate a human-like scenario to deduct relevant clinical information for patient treatment and to test novel therapeutic opportunities. Porcine models are particularly ideal due to a comparable cardiovascular system and size. However, certain anatomic disparities have to be taken into consideration when evoked potential monitoring is used in animal models. We describe a non-invasive and reproducible set-up useful for different modalities in porcine models. We further illustrate hints to overcome multi-faceted problems commonly occurring while using this sophisticated technique. Our descriptions can be used to answer a plethora of experimental questions, and help to further facilitate experimental therapeutic innovation.

## Introduction

Modern evoked potential (EP) monitoring is capable of rapidly and accurately diagnosing dysfunction of central and peripheral nervous system. This technique tremendously increases safety and helps to guide different surgical procedures, like operations on the medulla [1], on intradural structures [1,2], open thoracic aortic repair or thoracic endovascular aortic repair [3]. Moreover, differentiated neurological diagnostics, e.g. in multiple sclerosis patients [4] as well as prognostication of outcome after global cerebral ischemia in cardiac arrest patients [5] is facilitated. Various modalities have to be distinguished:

- Motor Evoked Potentials (MEP)
- Somatosensory Evoked Potentials (SSEP)
- Visually Evoked Potentials (VEP)
- Auditory Evoked Potentials (AEP)

In this article we provide a detailed description for non-invasive, fast and reproducible EP monitoring in a swine model. We demonstrate feasibility and describe a possible set-up of transcranial MEP (tcMEP) as well as median and tibial nerve SSEP (m/tSSEP). We like to share this valuable knowledge with researchers who aim to use this fast and reliable tool for a huge variety of future experimental projects.

## Methods

Ethical approval for porcine experiments was obtained from Regierungspraesidium Freiburg (G-14/39 and G-16/139), which is a governmental institution. It is staffed by a board team of specialist veterinarians, experienced in animal experiments and supervision thereof. The study was carried out in accordance to the National Institutes of Health’s “Guide for the Care and Use of Laboratory Animals” and reporting complies with the ARRIVE guidelines [6]. All procedures were performed under general anesthesia. First, pigs were sedated with intramuscular ketamine [20 mg/kg] and midazolam [0.5 mg/kg]. Then general anesthesia was maintained with continuous infusion of propofol [4–6 mg/kg/h]. In addition to total intravenous anesthesia (TIVA) no neuromuscular blocking agents were used. For our set-up, ten German landrace-hybrid pigs were used with a weight ranging from 31–63 kg. Animals were obtained from Rein-Hof, Breisach, Germany and received a full health check upon arrival to the animal care facilities by a veterinarian. The pigs were housed on solid floor covered with straw. The animals were fed a full standard diet by animal keepers until the evening before the experiment. Free access to water was enabled at any time. A veterinarian specialist, experienced in porcine experiment was present throughout the whole procedures. Upon completion of the experiments, animals were killed by intracardiac potassium injection under deep anesthesia. EP monitoring was performed using an ISIS IOM System (Inomed Medizintechnik GmbH, Emmendingen, Germany).

**Fig 1:**
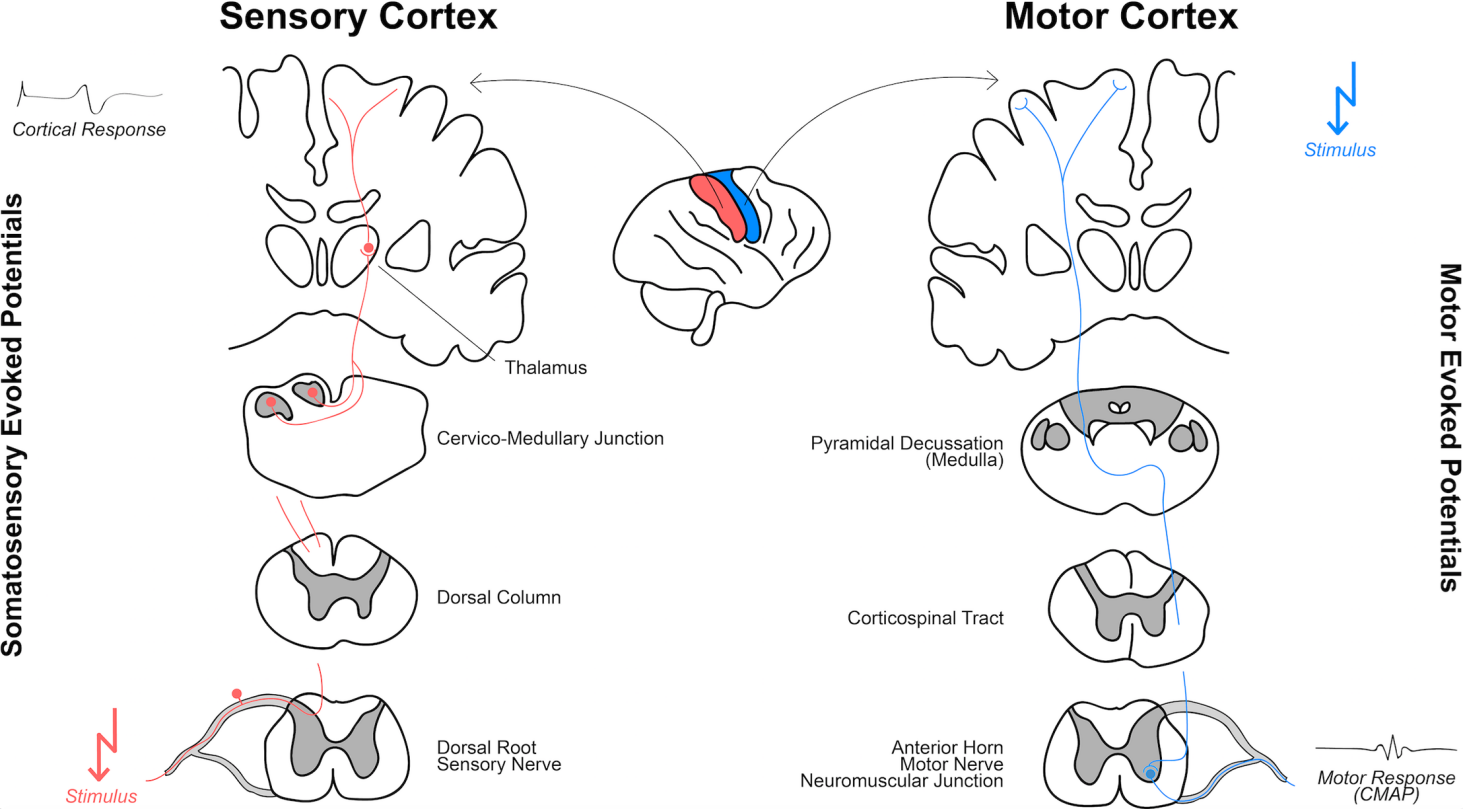
Principles of transcranial motor evoked potentials (tcMEP) and somatosensory evoked potentials (SSEP).

Generally, for tcMEP the motor cortex is stimulated transcranially while the resulting muscle response (compound muscle action potentials, CMAP) is evaluated (**Fig 1**). In contrast, SSEP monitoring consists of stimulation of a peripheral nerve which results in a cortical activity with a distinct pattern and latency (according to the respectively stimulated nerve). AEP and VEP represent a subclass of sensory EP, whereas auditory or visual stimuli are generated and potentials are measured at the respective cortical area [4].

EP monitoring systems are composed of a stimulating output, a recording input, an amplifier and a computing device. Importantly, an electrical grounding is absolutely necessary to prevent interfering signals from the power supply system. Commonly for SSEP monitoring, two stimulating subdermal needle electrodes are needed in close proximity to the nerve of interest while we use two cortical cork-screw electrodes for signal recording near the somatosensory cortex (referring to positions Cz/Fz in the international 10–20 system of human EEG). For tcMEP monitoring, two stimulating subdermal cork-screw electrodes are placed above the motor cortex (referring to positions C3/C4) and CMAP is recorded by placing two needle electrodes into the muscle of interest. Additionally one neutral electrode should be inserted on the trunk to improve signal quality.

### Transcranial MEP

**Fig 2:**
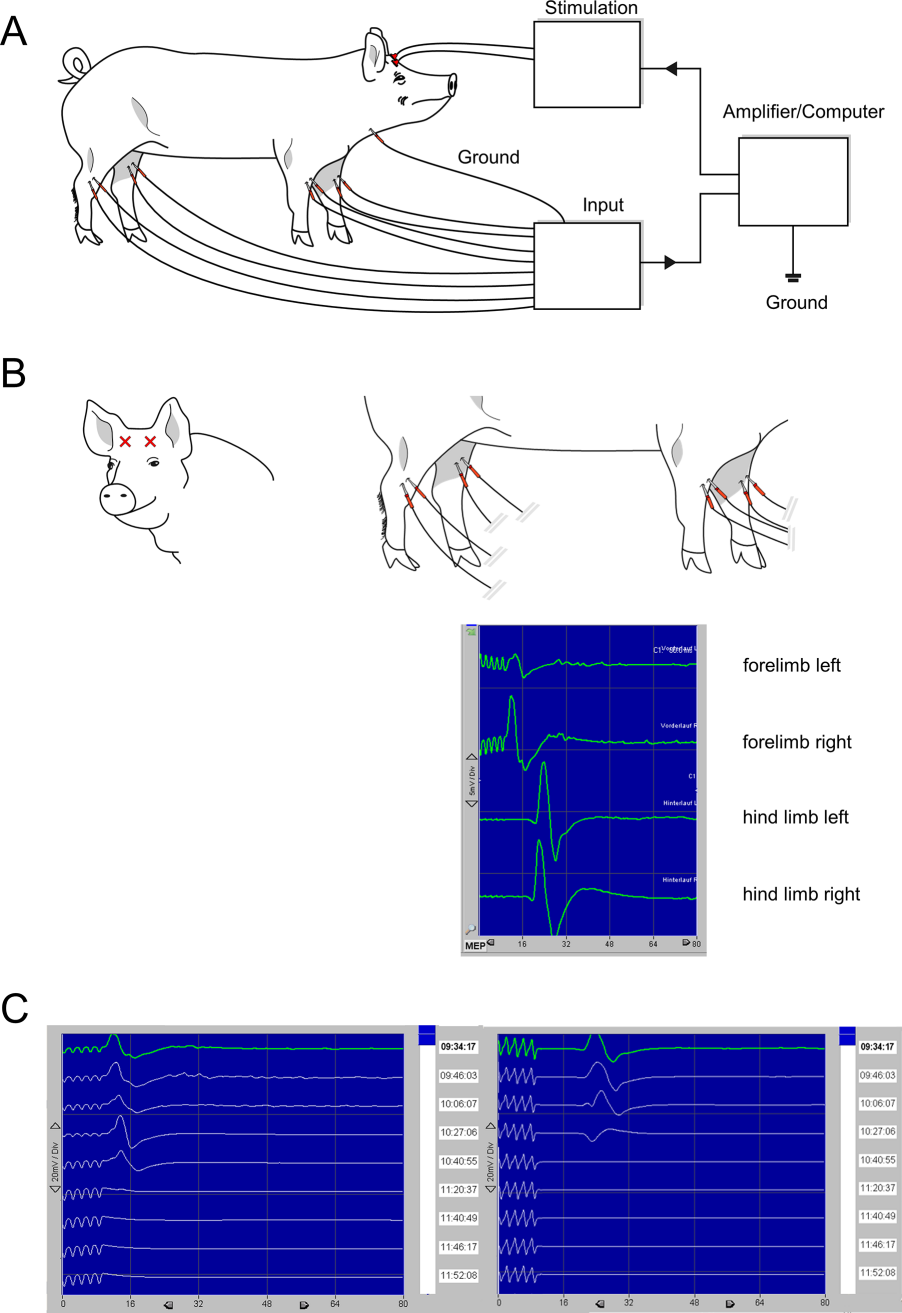
Measurement of transcranial motor evoked potentials (tcMEP) and resulting muscle response (CMAP). A) Schematic set-up of tcMEP. B) Electrode placement of the stimulation electrodes on the head (C3/C4 position) and recording electrodes on the forelimb and hind limb with exemplary CMAP (left and right side). C) Loss of CMAP on right forelimb and hind limb during experimental thoracic aortic procedure [6].

Parallel to an imaginary line between the ears and about 3–4 cm towards the eyes (**Fig 2**), two stimulating cork-screw electrodes are placed percutaneously at a distance of approximately 5 cm (in some cases a small skin incision may be helpful) in the area of the motor cortex (referring to the C3/C4 position in the international 10–20 system of human EEG). Recording electrodes of tcMEP were placed parallel into the extensor carpi radialis muscles or the triceps muscles at the left and right forelimb accordingly and into the tibialis cranialis muscles next to the tibia at the left and right hind limb at a distance of 3–4 cm (electrode length: 40 mm). Before the first measurement of EPs, a review of electrodes’ impedances should be performed to ensure the correct position in the muscle and the correct signal transduction from the electrodes to the computing device. We recommend impedance smaller than 2 kOhm for all tcMEP recording electrodes. During the experimental procedure the measurement of the impedance may be repeated in case of suspicion of accidental electrode malposition. Our experiences show that stimulation should be performed with 5 impulses with a width of 500 μs. A current of 130–190 mA is typically needed according to our experience. If there is no usable signal answer using these settings, there is the possibility to stimulate with 5 impulses in an alternating way with 1000 μs width. We do not recommend the use of 8 impulses for stimulation due to the coverage of the signal answer at the fore limb by the prolonged stimulation.

### Median and tibial nerve SSEP

**Fig 3:**
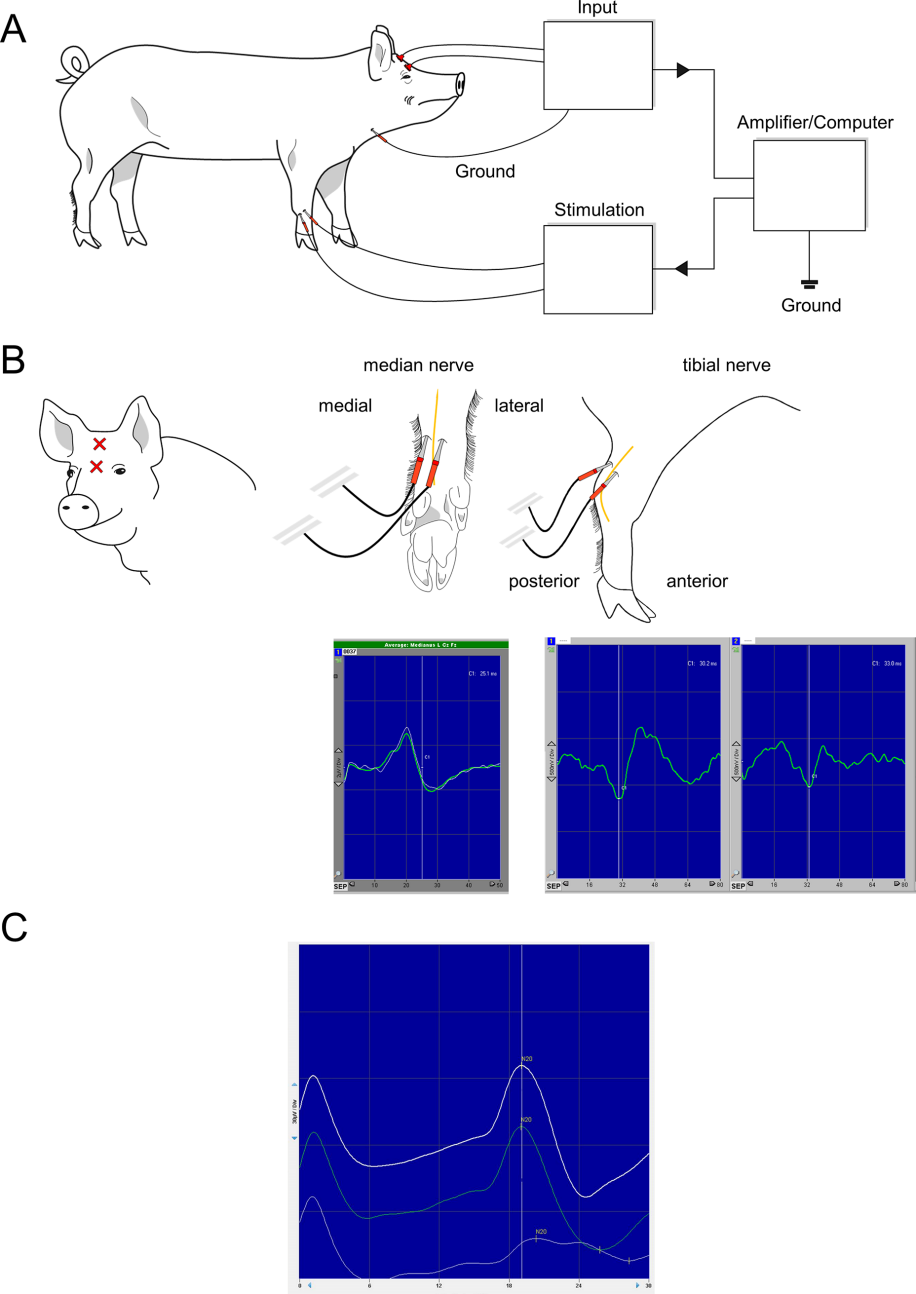
Measurement of somatosensory evoked potentials. A) Set-up of median nerve somatosensory evoked potentials (mSSEP). B) Electrode placement of the recording electrodes on the head (Cz/Fz position) and stimulating electrodes at the forelimb (median nerve [yellow]) and hind limb (tibial nerve [yellow]) (view from medial to lateral). Exemplary measurement of median (N20) and tibial nerve (P40) evoked potentials correspondingly. C) N20 potential at baseline (upper curve), prior to cardiac arrest (middle curve) and after induction of cardiac arrest (bottom curve).

SSEP stimulating electrodes (required electrode length 20 mm, distance from proximal to distal electrode approx. 3–4 cm) were percutaneously inserted paranerval on both sides of the tibial nerve at the hindlimb or in proximity to the median nerve at the forelimb (for anatomy see **Fig 3**). For SSEP recording the cork-screw electrodes were positioned in an imaginary line between neck and nose at a distance of 5 cm (referring to position Cz/Fz in the human 10/20 system).

Alternating tSSEP monitoring of the left and right side can be performed with a time delay of 80 ms by most commercial EP computers (e.g. ISIS IOM by Inomed). Although rectangular stimulating current is to be adjusted to a visible motor response, 25–35 mA are typically needed according to our experience. Stimulation frequency should be set to 4.7 Hz with a negative pulse form and a width of 200 μs. SSEP channels are band-passed from 5–600 Hz. Automatic detection and course of N20 (mSSEP) and P40 (tSSEP) latency and amplitude can be processed by EP computers, however averaging of at least 200 impulses is important which should be performed automatically. Describing potentials, “N” refers to negative (upward wave) and “P” to positive potentials (downward wave) according to international nomenclature.

## Results

Measurement of tcMEPs and SSEPs was feasible in all animals. Typical latencies and amplitudes of tcMEPs on the forelimb were 18.1±3.2 ms and 4.1±1.6 μV, and on the hind limb 28.5±3.9 ms and 2.3±1.5 μV respectively. SSEPs upon median nerve stimulation were 19.9±0.3 ms in latency and 4.4±0.8 μV in amplitude, while tibial nerve stimulation resulted in SSEPs at 30.3±1.4 ms in latency and 1.1±0.5 μV in amplitude (see **Table 1**).

**Table 1:** Table of tcMEP latencies and amplitudes on the forelimb and on the hind limb as well as latencies and amplitudes of median and tibial SSEPs.

**Animal No.**	**tcMEP Forelimb**			**tcMEP Hind limb**			**mSSEP**		**tSSEP**		
	Latency [ms]		Amplitude [μV]	Latency [ms]		Amplitude [μV]	Latency [ms]	Amplitude [μV]	Latency [ms ]		Amplitude [μV]
**1**	14.3		8.0	27.6		5.0	19.6	6.1	29.5		2.5
**2**	16.8		5.0	25.1		3.0	19.6	4.3	31.1		1.0
**3**	20.8		2.5	30.2		0.7	20.3	3.9	29.5		1.1
**4**	21.1		3.5	33.1		1.0	20	4.2	30.0		0.6
**5**	14.7		3.5	28.2		3.0	19.8	5.2	32.7		0.7
**6**	15.8		5.0	23.8		3.5	20.1	3.6	32.0		1.0
**7**	17.5		2.5	27.6		1.5	20.4	4	30.7		1.0
**8**	14.8		3.5	22.2		3.5	20.1	4.1	30.2		0.9
**9**	22.7		2.5	33.9		0.8	19.5	3.2	30.3		0.7
**10**	22.3		4.6	33.6		0.5	19.3	5.1	27.2		0.7
**Mean± SD**	18.1±3.2		4.1±1.6	28.5±3.9		2.3±1.5	19.9±0.3	4.4±0.8	30.3±1.4		1.1±0.5

## Discussion

EP monitoring represents a modern and powerful tool in neurological diagnostics and perioperative medicine. Here, we demonstrate a) successful implementation of state-of-the-art EP techniques into an experimental porcine model, b) explain ways to correctly place electrodes into the porcine anatomy for standard tcMEP and SSEP monitoring, and c) give hints towards interpretation and troubleshooting of EP monitoring in an experimental porcine model.

Despite previously published invasive approaches to tcMEP and SSEP modalities used in pig models [6–14], no description of a non-invasive and highly reproducible set-up including correct anatomical electrode placements and porcine-specific considerations exist in the literature so far. Thus, elaborate work is required to get acquainted with EP monitoring and review of porcine anatomical structures. In most cases trouble using EP monitoring occurs due to inappropriate settings, which highlights the necessity of appropriate know-how (see **Table 2**) [15]. We like to share our experiences with EP monitoring from our experimental porcine models, specifically enabling researchers in survival experiments to use EPs due to reduced invasiveness.

**Table 2:** Pitfalls of EP monitoring with problem, resulting presentation and potential solution.

**Problem**	**Presentation**	**Solution**
Incorrect electrode placement	No or variable response	Check Impedance of recording electrodes, Reposition electrodes, check distance in between electrodes [17],
Insufficient stimulating current	No or decreased muscle contraction, see **Fig 4A**	Increase stimulating current [18]
Complete neuromuscular block during MEP	No or decreased muscle contraction during MEP (note that SSEP monitoring is feasible on neuromuscular block), see **Fig 4B**	- Use quantitative and qualitative relaxometry - Wait for recovery of neuromuscular function or use pharmacological reversal agents [15,19]
Interference with power supply network	Concordant waves of 50 Hz, see **Fig 4C**	Check grounding and/or change power plug [15,20,21]
“Noise”	No distinct potentials / artifacts in signal, see **Fig 4D**	- Try to protect electrodes and twist electrode wires [22] - Try muscle relaxation for SSEP monitoring [18] - Make sure to use total intravenous anesthesia (e.g. propofol) [15,19] - Check Impedance of recording electrodes
Stimulation time too long	Potentials in tcMEP not visible on the fore limb, see **Fig 4E**	Avoid eight or more impulses or alternating stimulation with 1000 μs interval
Inversion of wave form	Negative SSEP potential downward or positive potential upward	Exchange position of electrodes on input box [17]
Recording of small electrical signals	Small amplitudes	- Check Impedance of recording electrodes and correct position of recording electrodes - Avoid volatile anesthetics [15,19,23] - Maintain body temperature [23] - Explainable by pathologic state?

**Fig 4:**
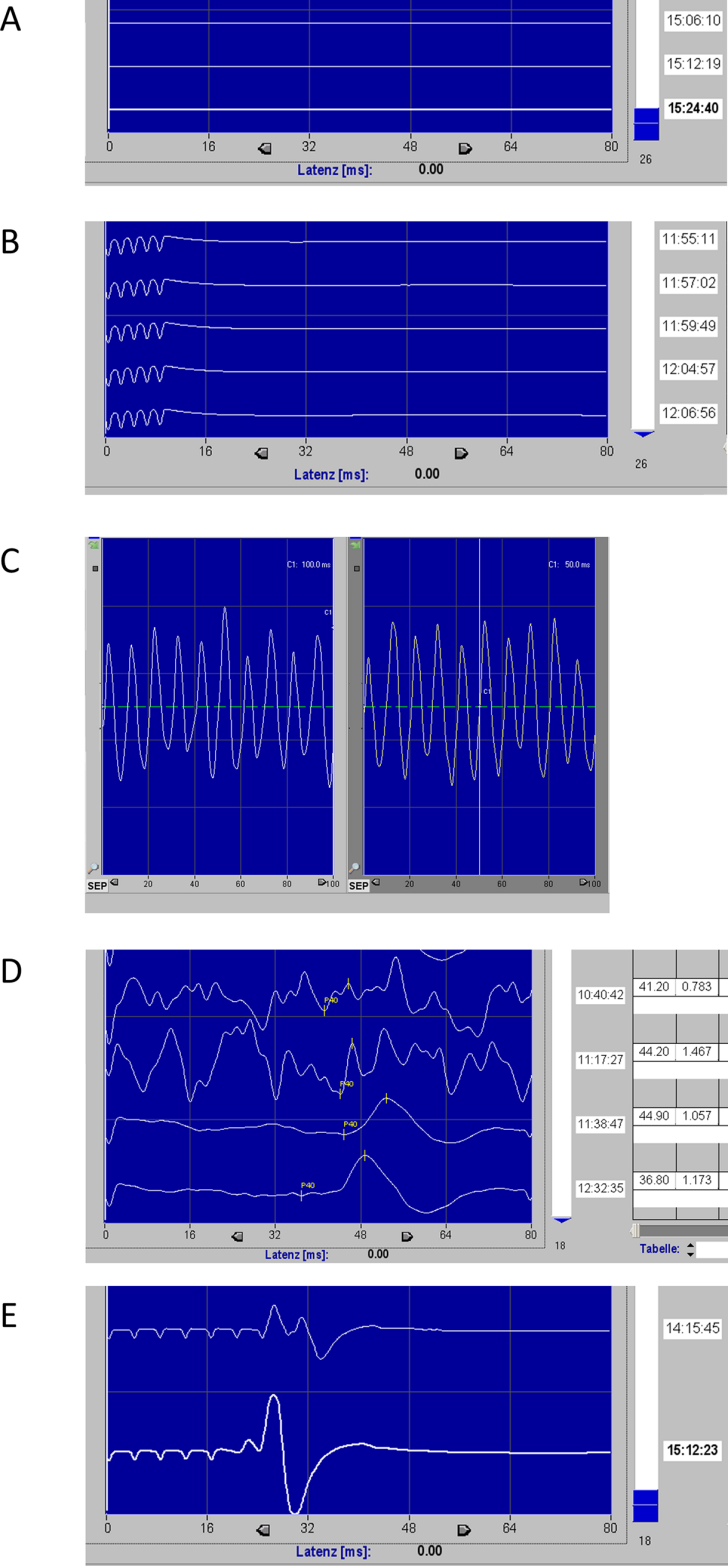
Screenshots of several problems during EP monitoring. A) Insufficient stimulating current or incorrect position of stimulation electrodes. B) Complete neuromuscular block during MEP. C) Interference with power supply network. D) Incorrect recording electrode placement, often recognizable via measurement of impedance. Good signal quality after correction of electrode placement in the two lower curves. E) Five stimulation impulses with signal answer directly after stimulation.

Certain disparities have to be considered when porcine EPs are compared to human EPs. While anatomical structures vary, latencies of electrical stimuli change due to shorter height of pigs requiring stimulation adjustments. Furthermore, no standardized 10/20 system for EEG exists to pinpoint cerebral structure, thus we reviewed porcine neuroanatomy to find corresponding regions (see Figs 2 and 3). Importantly, due to the smaller size of the pig’s brain, only small changes in electrode position on the head may result in insufficient measurements or stimulation of tcMEPs. Since the smaller size of the pigs’ muscles, it may be difficult to place the two recording electrodes of tcMEPs in the same muscle. However, this is very important to avoid the phenomenon of “jumping muscles” due to false negative changes in tcMEP signals [24]. Based on our experience in EP monitoring we used settings from human EP monitoring with low current and increased current until visible stimulation signals occurred (see **Fig 4A**). In contrast to humans, pigs exhibit a significantly larger skull diameter: Sauleau et al. report in the tomography study average skull thickness of 15–22 mm varying with age [25], being 3–4 times as thick as in humans. Puschel et al described currents of 200 mA for MEP stimulation on the scalp of the pig [14]. Owen et al used currents of 25–55 mA with subdermal needle electrodes [12]. In our setting (cork-screw electrodes, five stimulation impulses with a width of 500 μs) typically a current of 130–190 mA is needed to generate relevant signals.

In human SSEP monitoring it is common to read signals at different anatomical regions (e.g. C2 or Erb’s point in mSSEP), thus enabling distinct localization of pathology [26]. We have found this rather complex to implement in pigs due to lack of corresponding anatomical structures and do not regard this to be essential for most problems in experimental pig models.

When interpreting tcMEPs and SSEPs it is important to keep in mind that absolute values of SSEP amplitudes should only be compared among different pigs using great caution due to inevitable variation in electrode positioning. Rather, relative description of amplitudes (pre and post intervention) can render reasonable comparative character. Immanent to the method of EP are consideration of influentials and cofounders [15,16]: Type of anesthesia can significantly affect EP recording. Thus, total intravenous anesthesia (e.g. using propofol) should be preferred over volatile anesthetics whenever possible [15,19]. On the one hand, use of muscle relaxants can increase signal quality in SSEP due to reduction of noise, however tcMEPs cannot be securely read during complete neuromuscular block. Residual neuromuscular block can be excluded by using qualitative and quantitative relaxometry. In our experience intramuscular anaesthesia induction can interfere with tcMEP derivation as well, depending on the admission location. Changes in hemodynamics and body temperature should moreover be considered as major influentials and ideally be kept constant during EP monitoring. Electrocautery, surgical manipulation and electrical power systems further elicit interfering signals. Nevertheless, we cannot deny that a certain degree of experience in neurophysiology and knowledge in human EP monitoring should be acquired to correctly use this tool and distinguish these artifacts from real changes.

## Conclusions

We present a methodological description for performing and interpreting non-invasive EP monitoring in porcine models. Importantly, including EPs into an experimental protocol or choosing EPs as endpoint, provides a powerful tool for gaining insight and helps to delineate important online information on the neurological system. To the best of our knowledge we are the first study showing a successful, subcutaneous electrode approach for both SSEPs and MEPs in a porcine set-up resulting in high reliability, easy to perform and high reproducibility.

## Supporting information

S1 File Original data set. Table of tcMEP latencies and amplitudes on the forelimb and on the hind limb as well as latencies and amplitudes of median and tibial SSEPs.
(PDF)
