# Supplementary material for: Somatosensory and transcranial motor evoked potential monitoring in a porcine model for experimental procedures
Source: PLoS One. 2018 Oct 8;13(10):e0205410. doi: 10.1371/journal.pone.0205410 (PMC6175523; doi:10.1371/journal.pone.0205410)
Supplement: S1 File — Table of tcMEP latencies and amplitudes on the forelimb and on the hind limb as well as latencies and amplitudes of median and tibial SSEPs. (PDF) [file pone.0205410.s001.pdf]

Supporting Information

Original data set

Table of tcMEP latencies and amplitudes on the forelimb and on the hind limb as well as latencies and amplitudes of median and tibial SSEPs.

**Animal No.**	**tcMEP Forelimb**		**tcMEP Hind limb**		**mSSEP**		**tSSEP**	
	Latency [ms]	Amplitude [μV]	Latency [ms]	Amplitude [μV]	Latency [ms]	Amplitude [μV]	Latency [ms]	Amplitude [μV]
**1**	14.3	8.0	27.6	5.0	19.6	6.1	29.5	2.5
**2**	16.8	5.0	25.1	3.0	19.6	4.3	31.1	1.0
**3**	20.8	2.5	30.2	0.7	20.3	3.9	29.5	1.1
**4**	21.1	3.5	33.1	1.0	20	4.2	30.0	0.6
**5**	14.7	3.5	28.2	3.0	19.8	5.2	32.7	0.7
**6**	15.8	5.0	23.8	3.5	20.1	3.6	32.0	1.0
**7**	17.5	2.5	27.6	1.5	20.4	4	30.7	1.0
**8**	14.8	3.5	22.2	3.5	20.1	4.1	30.2	0.9
**9**	22.7	2.5	33.9	0.8	19.5	3.2	30.3	0.7
**10**	22.3	4.6	33.6	0.5	19.3	5.1	27.2	0.7
**Mean± SD**	18.1±3.2	4.1±1.6	28.5±3.9	2.3±1.5	19.9±0.3	4.4±0.8	30.3±1.4	1.1±0.5
